# Integrative rDNAomics—Importance of the Oldest Repetitive Fraction of the Eukaryote Genome

**DOI:** 10.3390/genes10050345

**Published:** 2019-05-07

**Authors:** Radka Symonová

**Affiliations:** Faculty of Science, Department of Biology, University of Hradec Králové, 500 03 Hradec Králové, Czech Republic; radka.symonova@gmail.com

**Keywords:** nuclear rDNA, rRNA, GC-content, secondary structure, nucleolus

## Abstract

Nuclear ribosomal RNA (rRNA) genes represent the oldest repetitive fraction universal to all eukaryotic genomes. Their deeply anchored universality and omnipresence during eukaryotic evolution reflects in multiple roles and functions reaching far beyond ribosomal synthesis. Merely the copy number of non-transcribed rRNA genes is involved in mechanisms governing e.g., maintenance of genome integrity and control of cellular aging. Their copy number can vary in response to environmental cues, in cellular stress sensing, in development of cancer and other diseases. While reaching hundreds of copies in humans, there are records of up to 20,000 copies in fish and frogs and even 400,000 copies in ciliates forming thus a literal subgenome or an rDNAome within the genome. From the compositional and evolutionary dynamics viewpoint, the precursor 45S rDNA represents universally GC-enriched, highly recombining and homogenized regions. Hence, it is not accidental that both rDNA sequence and the corresponding rRNA secondary structure belong to established phylogenetic markers broadly used to infer phylogeny on multiple taxonomical levels including species delimitation. However, these multiple roles of rDNAs have been treated and discussed as being separate and independent from each other. Here, I aim to address nuclear rDNAs in an integrative approach to better assess the complexity of rDNA importance in the evolutionary context.

## 1. The Eukaryotic rDNAome

**Figure 1:**
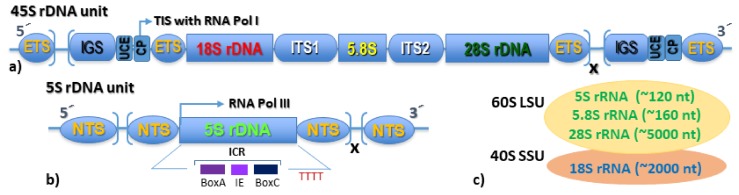
Brief guide to eukaryotic rDNAome—the genomic organization of the rDNA loci. (**a**) Structural organization of the 45S rDNA gene cluster (or rRNA transcription unit); the repeating or single clusters of rDNA can be found scattered throughout genome, they form the precursor pre-rRNA since ribonucleases remove spacers and release separate rRNA molecules in nucleolus—the site of ribosome biogenesis to polysome ribosome formation; (**b**) Structural organization of the 5S rDNA unit (the 5S rDNA can be also dispersed in the genome in many species); (**c**) 80S eukaryotic ribosome composed of the large subunit (LSU) and the small subunit (SSU) with outlined rRNAs. CP—core promoter, ETS—external transcribed spacer, ICR—internal control region, IE—internal element, IGS—intergenic spacer, ITS1, ITS2—internal transcribed spacer 1 and 2, RNA Pol I and III—RNA polymerase I and III, LSU—large (ribosomal) subunit, nt—nucleotides, NTS—non-transcribed spacer, SSU—small subunit, TIS—transcription initiation site, TTTT—polyT transcription termination site, UCE—upstream control element.

**Figure 2:**
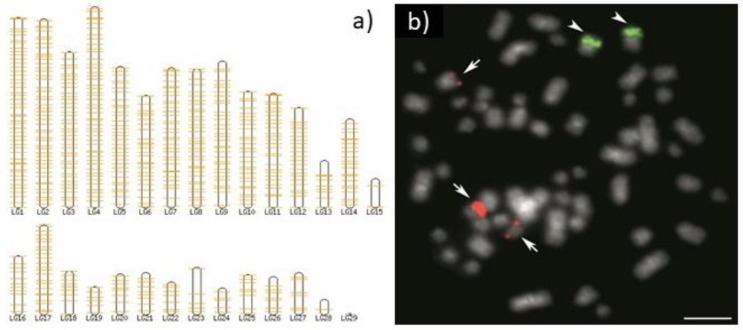
Comparison of two approaches of localization of rDNA on linkage groups and chromosomes in an ancient non-teleost ray-finned fish, spotted gar *(Lepisosteus oculatus*). (**a**) An in silico approach of visualization of the genomic position of rDNA loci utilizing the Ensembl genome browser tool BioMart to map 5S rDNA on linkage groups (LGs); (**b**) molecular cytogenetic localization of 5S (green, arrowheads) and 28S rDNA (red, arrows) on chromosomes by means of *fluorescence* in situ hybridization (FISH). Bar equals 5 μm (From [27], online Supplementary Material). This comparison shows the sensitivity of the in silico approach. The method enables detection of a single 5S rDNA molecule. It is possible to visualize dispersed molecules across the genome and their pseudogenes in this case. The FISH approach is limited only to huge clusters of accumulated rDNAs and has been utilized for decades particularly in cytotaxonomy in fishes, where most other cytogenetic markers work poorly. Both approaches have their own importance and justification and limits of their mutual interconnection at the current level of genomic data quality—chromosome pairs have not yet been assigned to their corresponding LGs in still too many of the sequenced species. More examples of 5S rDNA localization across LGs are available in Supplementary Materials Figures S1–S4.

RNA is essential for information flow from DNA to protein being the dominant macromolecule in protein synthesis [1]. Of the major RNA types, mRNA, tRNA, rRNA, and numerous short non-coding snRNAs, our focus here is on the nuclear rRNA encoded by ribosomal DNA (rDNA), i.e., by rRNA genes. In eukaryotic cells, up to 80% of RNA synthesis belongs to rRNA transcription indispensable to the preservation of ribosome biogenesis and protein synthesis [2]. There are about 1.5–3 million ribosomes per eukaryotic cell [3]. Hence, ribosome biogenesis consumes a tremendous amount of cellular energy and rRNA synthesis is tightly linked to cell growth and proliferation, and as such, it is responsive to general metabolism and environmental challenges [4]. In Eukaryotes, rRNA genes consist of several distinct multigene families tandemly arrayed as repeats composed of tens to hundreds or even thousands of copies. Beside two mitochondrial rRNAs, i.e., the 12S and 16S rRNA in eukaryotes, there are two fractions of nuclear rDNAs—a large, nucleolus-forming 45/47S rDNA unit and a substantially smaller extra-nucleolar 5S rDNA (Figure 1). Both the 45S and 5S rDNAs are organized into clusters of repeats often enabling their cytogenetic visualization on chromosomes [5]. The 5S rDNA can also (co)exist scattered separately within the genome e.g., in the spotted gar as shown in Figure 2a and in other organisms in S1–S4. In budding yeast, the rDNA has a very peculiar organization—the 5S rDNA unit is present in the intergenic spacers (IGSs) of the 45S rDNA and thus, alternating with 45S rDNA units [6]. The coding rDNA sequence is highly conserved among eukaryotes, while the IGSs (Figure 1) that separate the proper units of the 45S rDNA cluster can differ in length and sequence. In budding yeast, where rDNAs are particularly well-described, IGSs contain three unique elements that are common: an origin of replication, a replication fork blocking site and a promoter that directs the synthesis of noncoding transcripts [7]. In mammals, IGSs contain regulatory regions called UCE (upstream control element), CP (core promoter) and T (termination of transcription site) [8]. Only a fraction of the numerous rDNA copies is transcribed into rRNA. The non-transcribed rDNA copies are extremely important for integrity of the entire genome [7]. In yeast, strains with artificially reduced rDNA copy numbers became sensitive to DNA damage by chemicals and ultraviolet light. This sensitivity further increased as the number of rDNA repeats decreased [7]. In rats, mice, and clawed frog *Xenopus*, the IGS contains one or more RNA polymerase I (Pol I) promoters with high homology to the core region of the main rDNA promoter [9]. Transcripts originating from spacer promoters are co-directional with pre-rRNA synthesis and enhance transcription from the main rDNA promoter, possibly by releasing Pol I [10,11]. Intergenic spacers rRNA have a crucial function in rDNA silencing. In mice, intergenic transcripts originating from a promoter located approximately 2 kb upstream from the pre-rRNA start site are processed into a heterogeneous population of 150–250 nucleotide RNAs, dubbed promoter RNA (pRNA) as their sequence matches the rDNA promoter [6,8,9,11].

## 2. The Multifaceted Nucleolus

Multiple copies of rRNA gene clusters form nucleolar organizer regions (NORs), the NORs, around which nucleoli are built in the interphase nucleus. The nucleolar 45S rDNAs are transcribed by RNA polymerase I into rRNAs, further processed and assembled with ribosomal proteins into ribosomes [12]. Nucleoli form at the end of mitosis and persist until the onset of the next mitosis. Active nucleoli, where the pre-rRNA transcription takes place, can be visualized in nuclei by silver impregnation, the argyrophilic Ag-NOR staining [13]. From the ultrastructural viewpoint, avian and mammalian nucleoli contain three components (fibrillar centers, dense fibrillar component, and granular component) and differ from all other eukaryotes that possess bipartite nucleoli (i.e., a network of fibrillary strands embedded within granules) [14]. Interestingly, both types of nucleolar arrangement occur among living reptiles: a bicompartmentalized nucleolus in turtles and a tricompartmentalized nucleolus in lizards, crocodiles, and snakes [15]. From the functional viewpoint, the nucleolus was long regarded as a mere ribosome-producing factory. However, during recent decades numerous and crucial non-ribosomal roles were described for the nucleolus [4]. Now, there is a still growing body of evidence that the nucleolus is central to cellular processes as varied as stress response, cell cycle regulation, RNA modification, cell metabolism, and genome stability and integrity [7]. All organisms sense and respond to stressing conditions by downregulating the transcription of rDNA to rRNA and ribosome biogenesis as these processes are extremely energy-consuming [4].

## 3. The Nucleolus Forming 45S rDNA

The 45S rDNA transcription unit forms a precursor pre-rRNA consisting of 18S, 5.8S, and 28S rRNAs separated by two internal transcribed spacers (ITS1, ITS2) that are removed during the rRNAs maturation process. The entire unit is further delimited by external transcribed spacers (ETS). Intergenic spacers (IGS; Figure 1a) separate each such unit with both of its sides bearing important regulatory elements [16]. This nomenclature applies to the Animal Kingdom. In plants, there is a 25S rDNA gene (instead of the 28S rDNA) within the large nucleolar rDNA multigene family 35S rDNA (instead of the 45S rDNA [17]). In unicellular organisms, where budding yeasts are the most important model system, the 35S rDNA consists of 25S, 5.8S, and 18S together with the 5S rDNA localized into the intergenic spacer within the 35S rDNA [18]. By addition of some 50–60 ribosomal proteins, the 25S/28S, 5.8S, and 5S rRNAs are fashioned into the large ribosomal subunit, 60S LSU. The 18S rRNA associates with 30–40 ribosomal proteins to form the small ribosomal subunit, 40S SSU (Figure 1c). Molecular cytogenetic localization of the rDNAs 28S rDNA fraction of the 45S rDNA unit on chromosomes is shown on Figure 2b.

The importance of nucleolus-forming rDNA and its proper functioning can be seen in the phenomenon of nucleolar dominance. Nucleolar dominance (or a nucleolus under-development due to expression of rDNA from just one parent) is a dramatic disruption in the formation of nucleoli and epigenetically controlled silencing of 45S rDNA in one of the progenitors in an interspecies hybrid. It is characteristic of some plant and animal distant hybrids and represents an example of a non-mammalian maternal imprinting of 45S rDNA [19,20]. Among animals it has been so far evidenced in details in intra-generic hybrids of *Xenopus* [20,21], in inter-generic hybrids of cyprinid fish [22,23] and in two lines of mouse-human somatic hybrids, where the human ribosomal genes were repressed, and only mouse ribosomal genes were expressed [24]. In the species *Drosophila melanogaster*, a special example of allelic inactivation resembling nucleolar dominance exists [25]. *D. melanogaster* carries its rDNA array on the X and on the Y chromosome [26], but the entire X chromosome rDNA array is normally silenced in *D. melanogaster* males, while the Y chromosome rDNA array is dominant and expressed [25].

## 4. The Extra-Nucleolar 5S rDNA

The 5S rDNA is much shorter and far less complex within its tandem array structure in comparison with the 45S rDNA. The 5S rDNA consists of a highly conserved sequence of about 120 bp coding for the 5S rRNA and including following functional elements: Box A, IE, Box C [28]. This transcribed sequence is separated at both of its ends from other transcriptional units by a highly variable non-transcribed spacer (NTS; Figure 1b). The participation of 5S rRNA on the ribosome structure is shown on Figure 1c. The 5S rRNA enhances protein synthesis by stabilizing the ribosomal structure and the peptidyl transferase activity, and potentially transmits and coordinates functional centres of the ribosome [29,30]. Two ways for visualization of 5S rDNA sites in silico on linkage groups (LGs) utilizing genomic data and employing methods of molecular cytogenetics on chromosomes are shown in Figure 2. More examples of in silico visualization of 5S rDNA on LGs are in the Supplementary Materials Figures S1–S4.

Two tissue and developmentally specific types of 5S rDNA exist in lower vertebrates, namely the somatic and the oocytes-specific ones. In fish, the oocyte type is lost during development completely (e.g., [31]). Whereas in a frog, it is lost largely [32]. The oocyte repeat comprises a 120 bp oocyte-type 5S rRNA gene placed within the few hundred bp long native AT-rich flanks, whereas the somatic repeat, i.e., a similar 120 bp somatic-type 5S rRNA gene is placed within native GC-rich flanks [33]. Instability of the oocyte 5S rRNA gene transcription complex contributes to the inactivation of the oocyte 5S rRNA gene during embryogenesis [34].

Moreover, in bony and cartilaginous fish, two types of co-occurring 5S rDNA can be distinguished. These probably paralogous type I and II were described in bony fish [35,36] and in elasmobranchs [28]. These types differ by the length and the sequence of their NTS region whereby the longer version is designated as type II [28]. Three functional variants of 5S rRNA genes exist in all life stages of common sea urchin *Paracentrotus lividus* [37]. Systematic study of 5S rDNA sequence diversity in 97 metazoan species [38] describe several paralogous 5S rDNA sequences in 58 of the examined organisms and a flexible genome organization of 5S rDNA in animals. This study also describes three different types of termination signals and variable distances between the coding regions and the typical termination signal. Importantly, a consensus sequence and secondary structure of metazoan 5S rRNA is presented in this study [38], which can be very useful in more detailed future studies of both 5S rDNA and rRNA.

## 5. Copy Number Really Matters

Gene duplication is an important and frequent evolutionary process [39] and the resulting copy number variation (CNV) is the most frequent type of genetic variation per base pair in the population [40]. Although alteration of gene copy number or gene dosage has deleterious effects for a significant fraction of the genome, changes in dosage are well tolerated in many genes (reviewed by [41]). CNV of rDNA is highly studied in rDNAomics since it provides a mechanism for cellular homeostasis and for rapid and above all reversible adaptation [42,43,44]. Due to the tandem repetitive structure of rDNA, the repeat number can be easily reduced by homologous recombination among the repeats. However, there is a finely tuned ´gene amplification system´ compensating for these losses and another highly sophisticated system controlling the ´proper´ rDNA copy number [7]. Moreover, these systems are capable of linking external nutrients availability with rDNA copy number [45] that proves the role of rDNA in the cellular energy metabolism as described for nucleolus above. This illustrates how crucial the right copy number of rDNA is for each cell. Moreover, as uncovered at least in humans, the CNV of rDNA represents a novel and cryptic source of hypervariable genomic diversity with far-reaching global regulatory consequences [42]. However, we have accumulated only limited understanding of the immense importance of these seemingly passive and simple phenomena tightly linked with regulation of nuclear as well as mitochondrial genes expression [42] and probably with many more essential cellular mechanisms. There is an inconsistency in the quantification of rDNA copy number even in the human genome. One important study states that the rDNA copy number varies among healthy humans as a result of natural genetic diversity between 14–410 copies of the 45S rDNA unit per genome [41]. A more recent study reported that the number of rDNA repeats varies from 250 to 670 copies per diploid genome [46]. Therefore, data provided by Gibbons et al. [41,45] should be treated with caution as the low limit number of 14 copies has not been otherwise found in mammals. The genomes of higher eukaryotes harbour hundreds and thousands of copies and only prokaryotic genomes can carry fewer copies of ribosomal genes. Moreover, given that there are five pairs of clusters of ribosomal genes, located on five pairs of human acrocentric chromosomes (13, 14, 15, 21, 22), it is logically impossible that ten clusters could totally count 14 copies, i.e., just 1.4 copies per cluster. These obviously underestimated values can be explained as artifacts caused by a poor suitability of PCR-based techniques for the quantification of GC-rich moderate repeats used by Gibbons et al., 2014, 2015 [42,47] compared to more suitable nonradioactive quantitative hybridization (NQH) used by Chestkov et al., 2018 [46]. The reason is that rDNA is a specific region often forming non-canonical hairpin and loop structures and prone to oxidation in vivo and after extraction from cells [46]. Phenotypic effects of rDNA copy number were recently summarized by [48]. CNV of rDNA (loss as well as amplification) is linked to tumorigenesis [49,50].

A substantial intra-species CNV of rDNA was, among others, evidenced in a freshwater microcrustacean *Daphnia* [51]. rDNA copy number does change among tissues and during ontogenesis in multicellular organisms [31,52] and it is age-dependent in single-cell yeasts [7]. Substantial differences in rDNA CN and number of their sites on chromosomes have been repeatedly recorded in vertebrates and invertebrates on the inter-population and inter-species level of comparison [5]. Such genomic differences might also contribute to genome diversifications, reproductive barriers formation, speciation events and finally to an increased biodiversity, e.g., [53,54]. Numerous examples from fish cytogenetics show that the variation in rDNA repeats prove to be highly informative as it is subject to a more relaxed regulation than in higher vertebrates [55]. Here, traditional cytogenetics meet the currently booming genomics to mutual usage and benefit from each other. The Animal rDNA database currently contains 539 records on fish rDNAs, namely 5S rDNA in 417 species and 45S rDNA in 479 species [5]. However, a detailed analysis of rDNA sequence organization and variation and CNV on the molecular level exists only for a handful of fish species including both 5S and 45S rDNA of zebrafish [31,51], 5S rDNA and only partial 45S rDNA of pike [56], only 5S rDNA of tilapia [57], molecular organization of the 5S rDNA type II of elasmobranchs (i.e., sharks, rays, and skates [28]) and cichlids [58]. In *Drosophila* germline stem cells, rDNA copy number decreases during aging and this age-dependent decrease in rDNA copy number is transgenerationally heritable. However, young animals are capable of recovering the normal rDNA copy number [59]. The copy number obviously plays a functional role: in *Xenopus*: the somatic 5S rDNA has about 400 copies, while the oocyte 5S rDNA has about 20,000 copies [32]. Locati et al. [31] detected about 9000 5S rRNA genes in the zebrafish genome assembly GRCz10 [31] and Symonova et al. detected about 20,000 copies of 5S rRNA genes in the Northern pike *Esox lucius* and its congener *E. cisalpinus* [56]. However, the record holders are currently protists, namely ciliates: Oligotrichia and Peritrichia [60] and representatives of the ciliate group Spirotrichea - *Oxytricha nova* with about 200,000 rDNA copies [61] and *Stylonychia lemnae* with estimated 400,000 copies of rDNA [62]. The already mentioned single-cell ciliate protozoan *Tetrahymena* amplifies its rDNA 9000-fold during development of the somatic macronucleus [63]. Whereas the copy number of 45S and 5S rDNA units is tightly coupled in mouse and human [47], such a control is apparently missing in fish [52,56,64]. This fact together with the aforementioned difference in nucleolar organization between higher and lower vertebrates and also other genomic traits (e.g., genomic GC heterogeneity) indicate that another major evolutionary transition *sensu* [65] occurred in evolution from anamniotes towards amniotes. This huge copy number variation might be linked to (or might have resulted in) the heterogeneity in rRNA genes and their variants that had been considered a peculiarity of some plants. Only recently, this heterogeneity was proved also in animal ribosomal genes, including human and mouse, where variant rRNA alleles exhibit tissue-specific expression and ribosomes bearing variant rRNA alleles are present in the actively translating ribosome pool [66].

One special topic of the rDNA CNV is based on molecular cytogenetic localization of both 5S and 45S rDNAs using FISH. FISH with rDNAs represents one of the most important chromosomal markers particularly in non-model organisms and especially in cold-blooded vertebrates, where methods like R-banding do not yield any usable and reproducible pattern. For these reasons, a heavy body of literature on molecular cytogenetics of rDNA has accumulated (for plants [17], for animals [5]). Since rDNA was omitted from many genome sequencing projects due to issues with its assembling [50,67], any precise quantification of rDNA copies is mostly still impossible. On the other hand, the still increasing availability of long-read sequencing can overcome assembling issues and provides opportunity to link the numerous results from molecular cytogenetics with genomics as was successfully demonstrated in fish cytogenomics [54,56].

A very special chapter of the rDNAomics book deals with rDNA of eukaryotic microorganisms [67]. In their 2010 review, Torres-Machorro et al. present available information on both rDNA fractions from about hundred microbial eukaryotes and show an unexpected diversity in their genomic organization [68]. Later, Drouin and Tsang [69] focus their review of 5S rDNA in protists on adaptive potential of its organization. Microbial eukaryotic rDNAs may be coded alone, in tandem repeats, linked to each other or linked to other genes. They exist in the chromosome or extrachromosomally in linear or circular units and rDNA coding regions may contain introns, sequence insertions, protein-coding genes, or additional spacers [68]. The atypical structures of rDNA have been considered as exceptions. However, it is rather likely that these organisms have preserved variations in the organization of these versatile genes that may be considered as living records of evolution [68]. A huge step in establishing the functional significance of rDNA in evolution and in ecology of organisms has been performed in protists [60].

## 6. Overview of Important Facts about rDNA

The most import facts about rDNA can be summarized as follows: rDNA is ubiquitous and universal across Prokaryotes, Archaea, and Eukaryotes [70]. It has a high degree of functional and sequence conservation of rDNA genes [71]. At the same time, rDNA belongs to the most copy number-hypervariable genomic segments [42] and the tandemly repeated rDNA arrays are among the most evolutionary dynamic loci of eukaryotic genomes in terms of copy number. Due to its heavy transcription, repetitive structure, and programmed replication fork pauses, the rDNA is one of the most unstable regions in the genome [7,18]. Their high genomic copy number relative to other genes appears to be much larger than required, however, unlike protein-coding genes, rDNA cannot undergo additional rounds of amplification via translation when organisms require more rRNA transcripts [72]. Copy number of 45S units is balanced with that of the 5S rDNA in mouse and human [47] but not in fish, summarized by [5]. These multiple copies of rDNA evolve in a highly coordinated manner, through unequal crossing over and/or gene conversion, two mechanisms related to homologous recombination [73]. The rRNA gene repeats use a unique gene amplification system to restore the copy number after this has been reduced due to recombination [7]. The RFB (replication fork barrier) coordinates replication and recombination, and through the latter, mediates a possible increase in the number of rDNA repeats. rDNA loci are dynamic genetic elements, their copy number changes dynamically and transgenerationally yet is maintained through a recovery mechanism in the germline (for *Drosophila* see [59]). In plants, extensive variation can exist in both rDNA copy number and rRNA expression. Among maize inbred lines, thousands of genes co-regulate with rRNA expression, including genes participating in ribosome biogenesis and other functionally relevant pathways [74]. Not only the rDNA copy number [45] but also the rRNA expression variation is a valuable source of functional diversity that affects gene expression variation and field-based phenotypic changes [74]. The intra-genomic homogenization of rDNA mostly occurs through ‘concerted evolution’ [75]. rDNA also shows high rates of meiotic recombination [75,76] and rDNA sites are hotspots for genome rearrangements [77]. Copy number of rDNA arrays modulates genome-wide expression of hundreds to thousands of genes and subtle changes in rDNA copy number between individuals may contribute to biologically relevant phenotypic variation also in humans [78]. rDNA contributes to global chromatin regulation and thus to a balance between heterochromatin and euchromatin in the nucleus [79]. The enormous variation in the number of rDNA copies per eukaryotic genome correlates with genome size [80] and the copy number of the 45S rDNA fraction was shown to negatively correlate with mtDNA abundance [42]. Hence, rDNA copy number variation, CNV (“rDNA dosage”) is a major determinant of naturally occurring genome-wide gene expression variation in humans [42]. Ribosomal RNAs (rRNAs) account for up to 80% of all RNAs in eukaryotic cells [50]. Growth-activated rRNA synthesis may be mediated by the up-regulation of individual rDNA units, in addition to the activation of silent gene copies see e.g., Banditt et al. [81]. In mammals, 5S and 45S rDNA arrays are non-homologous, physically unlinked, transcribed by different RNA Polymerases and encode functionally interdependent RNA components of the ribosome [47]. Clusters of the 45S rDNA unit give origin to the nucleolus, the nuclear organelle that is the site of pre-45S rDNA transcription and ribosome biogenesis, see e.g., [8,82]. The rDNA contact map shows that 5S and 45S arrays each have thousands of contacts in the folded genome, with rDNA-associated regions and genes dispersed across all chromosomes [83,84]. Due to its highly repetitive nature, rDNA has been excluded from most mammalian genome-wide studies because of challenges associated with its analysis, and thus remains understudied. There is an unusual and universal GC richness of the 45S rDNA fraction in cold- as well as warm-blooded vertebrates (more details below) [72,84]. An extensive range of epigenetic modifications regulating rRNA genes transcription [67,85,86,87] results in only a mere subset of the multiple copies being transcribed however with far reaching implications for the entire genome (elucidation of the epigenetics of rDNA in sufficient detail would require a lot of research). On top of it, rDNA loci serve as a specialized niche for mobile elements [88].

rDNA units (so called ‘rDNA-like signal’) can be found scattered throughout the genome in humans [89]. These units can be described as follows: 1) highly degraded, but near full length, rDNA units, including both 45S and Intergenic Spacer (IGS), can be found at multiple sites in the human genome on chromosomes without rDNA arrays; 2) these rDNA sequences have a propensity for being centromere proximal; and 3) sequence at all human functional rDNA array ends is divergent from canonical rDNA to the point that it is pseudogenic. For this in fish, see Figure 2a and in other chordates see the Supplementary Materials Figures S1–S4.

rDNA represents a cryptic source of hypervariable genomic diversity with global regulatory consequences (ribosomal quantitative trait loci (eQTL)) in humans. The variation provides a mechanism for cellular homeostasis and for rapid and reversible adaptation [42,47].

## 7. GC Content of rDNA

The 45S rDNA gene clusters form the GC-richest genomic fraction particularly in Eukaryotes [90] with humans having 60%–80% GC in different parts of the rDNA [91], whereas the median genomic GC is 40.9% (NCBI, human genome assembly). This GC-richness is ascribed to the recombination rate based process known as GC-biased gene conversion [73]. On the other hand, some studies link the extremely high GC levels in rDNAs to particular requirements for stem-and-loop systems in rRNA that have an effect on the composition of the corresponding genes to thermal adaptation [92]. This line of explanations belongs to the Thermodynamic Stability Hypothesis attempting to account for the overall AT/GC heterogeneity in birds and mammals and the AT/GC homogeneity in the remaining vertebrates and the other Eukaryotes [90]. However, although Wang et al., 2006 showed support for such a thermal adaptation in Bacteria and Archaea, they did not find any for warm-blooded birds and mammals with only a slightly higher GC content of 18S (55.7%) versus cold-blooded fishes and amphibians with approximately 53.5% of GC. Their partitioning of the GC content across the 18S rRNA sequences into stem and loop regions demonstrated [93] that the differences are not concentrated in the paired stem regions as expected by Bernardi [90]. Interesting and relevant aspects of rDNA GC content exist in so-called expansion segments (ES) in 28S and 18S rRNA molecules [94,95]. Expansion of the 28S rRNA shows a clear phylogenetic increase, with a dramatic rise in mammals and especially in hominids. Here, a GC- or AU-biased expansion of rRNAs has developed in both plants and metazoans, with the GC-bias largely being preferred in extremely GC-rich ES of vertebrate 28S rRNA. This compositional bias towards GC is linked to potential roles of GC-rich rRNA during protein synthesis [96,97] and could contribute to the discussion whether the genomic GC content is driven by neutral versus selective processes. An interesting explanation of the universal GC richness of 45S rDNA comes from the GC biology—these multicopy genes should all be in a DNA region with a homogenous GC composition to allow concerted evolution and to prevent divergence through generations [98].

## 8. Phylogeny, Species Delimitation, and Secondary Structure of rRNAs—The Way How to Determine in Silico Whether Two Lineages Can Successfully Cross

**Figure 3:**
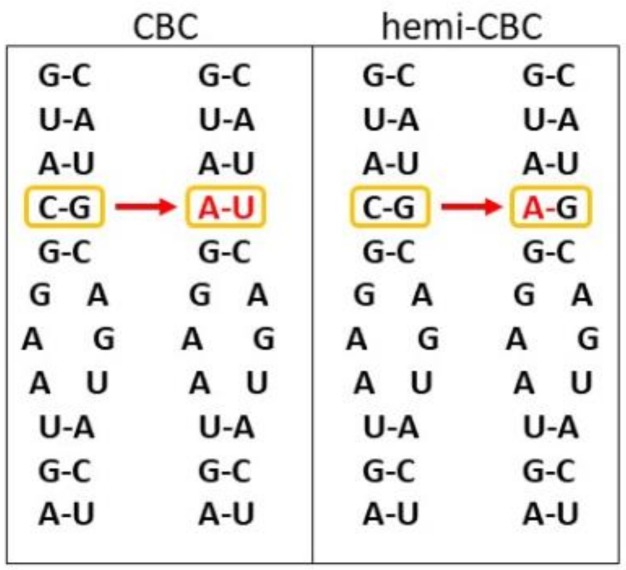
Visualization of compensatory base changes (CBCs) on a hypothetical internal transcribed spacer 2 (ITS2) secondary structure. The left panel depicts a conserved helix segment in which a CBC occurs, where both nucleotides of the pair underwent a mutation that resulted in retaining the paired nucleotide bond. The right panel shows a hemi-CBC, where only one nucleotide of the pair, i.e., C to A, underwent a mutation while the pair retained the nucleotide bond. Redrawn according to [106].

The ITS2 sequence already belongs to the most popular and well established phylogenetic and DNA barcoding markers [99]. The rDNA sequence and its corresponding rRNA secondary structure is one of the few universal features of life without any known case of horizontal transfer and above all, identifying the organism to a unique species, making it uniquely suited to assess phylogenetic relationships [100,101]. The secondary structure of the ITS regions is well known for a wide variety of eukaryotes and have been used to aid in the alignment of these sequences for phylogenetic comparisons [101]. The RNA sequence of the ITS2 possesses another special trait so far not fully examined, namely compensatory base changes (CBCs, Figure 3). CBCs are mutations occurring simultaneously on both sides of a nucleotide pair in the ITS2 secondary structure with retention of the paired nucleotide bond, whereas hemi-CBC is a mutation of a single nucleotide of the pair still retaining the bond [102]. CBC analyses have been primarily performed in fungi and plants [92,93,94]. This is the reason why the majority of literature references, including methods descriptions, are on plants (e.g., estimating structure-based phylogenetic trees from ITS2 data by [103,104,105]). CBCs analyses have been already successfully used to verify taxonomy of closely related species and to distinguish morphologically indistinct species in insects [102]. That shows the huge potential of mining for CBCs in ITS2 rRNA secondary structures also in the Animal Kingdom.

**Figure 4:**
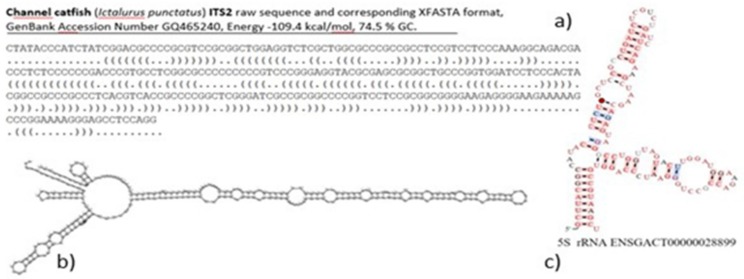
Text and graphic representation of prediction of rRNA secondary structure. (**a**) the Xfasta or "dot-bracket notation" way of representation of the ITS2 rRNA secondary structure of channel catfish, (**b**) visualization of the corresponding secondary structure of the sequence in (**a**) with one longest helix and four short helices, (**c**) stickleback, prediction of 5S rRNA secondary structure, Ensemble.

However, any detailed and particularly systematic survey of rRNA secondary structure in the Animal Kingdom is still in its infancy although it would be highly desirable in numerous areas of biology. Moreover, analyses of rRNA secondary structure could represent another intersection between rDNAomics based on molecular cytogenetics and on genomics since molecular cytogenetic studies frequently provide DNA sequences of rDNA fragments used in FISH experiments and further DNA sequences, in the meanwhile, became available in NCBI GenBank or could be retrieved from whole-genome datasets. Ideally, such integrative studies could contain cytogenetic results accompanied by details on rDNA/rRNA sequence and rRNA secondary structure as shown in Figure 4 to better explore any potential sequence polymorphism.

## 9. Concluding Remarks

The field of rDNAomics is extremely rapidly evolving further and has far-reaching implications for numerous areas of current biology and medicine. Medical aspects represent another crucial chapter of rDNAomics that already exceeds the scope of this review. On the other hand, being aware of this fact might help scientists from the area of fundamental research working on non-model organisms to provide justification of their work. There are numerous diseases associated with rDNA dysfunction, particularly cancer [39,40,61,66]. Ribosomopathies are diseases caused by abnormalities in the structure or function of ribosomal component proteins or rRNA genes, or other genes whose products are involved in ribosome biogenesis [107]. Not only sequence, but also copy number of rDNAs is of particular importance in cancer—human cancer genomes show a loss of copies, accompanied by global copy number co-variation [50]. Even more relevant is the fact that rDNA repeat instability coincides with predisposition to cancer, premature aging and neurological impairment in ataxia-telangiectasia and Bloom syndrome (Warmerdam and Wolthuis, 2018). Additionally, it was shown that cancers undergo coupled 5S rDNA array expansion and 45S rDNA loss that is accompanied by increased proliferation rate and nucleolar activity. Somatic changes in rDNA copy number can exceed 10-fold the naturally occurring copy number variation across individuals [49]. Malfunction of nucleoli can be the cause of several human conditions called nucleolopathies [108]. The nucleolus is being investigated as a target for cancer chemotherapy [109,110]. Moreover, rDNA copy number may be a simple and useful indicator of whether a cancer will be sensitive to DNA damaging treatments [50]. Hence, it is desirable to understand rDNA organization, function, and its impact on the entire nucleolus and other genes’ regulation (as briefly outlined here) in a broader evolutionary context.

## Supplementary Materials

The following are available online at https://www.mdpi.com/2073-4425/10/5/345/s1, Figure S1: *Tetraodon nigroviridis*, karyogram with labeled 5S rDNA loci (red arrowheads). Figure S2: Zebrafish (*Danio rerio*), complete karyogram with labeled 5S rDNA loci (pink lines). Figure S3: A tunicate sea squirt (*Ciona intestinalis*), C ≈ 0.2, the thirty-five 5S rDNA site detected on four chromosomes could be assigned to a linkage group. Figure S4: Human genome illustrates here the best assembled vertebrate genome, C ≈ 3.5. Genomic localization of annotated 5S rDNA showing 5S rDNA scattered throughout fish genomes visualized on karyograms of species assembled to the chromosome level (5S rDNA sequences were filtered using the BioMart tool and the Ensemble Genes Database version 92 from Ensembl.org version 92). For comparison, two model mammalian genomes (Hsa, Mmu) and one avian genome are shown.
