# Supplementary material for: Integrative rDNAomics—Importance of the Oldest Repetitive Fraction of the Eukaryote Genome
Source: Genes (Basel). 2019 May 7;10(5):345. doi: 10.3390/genes10050345 (PMC6562748; doi:10.3390/genes10050345)
Supplement: Supplementary file 1 [file genes-10-00345-s001.pdf]

Supplementary Material

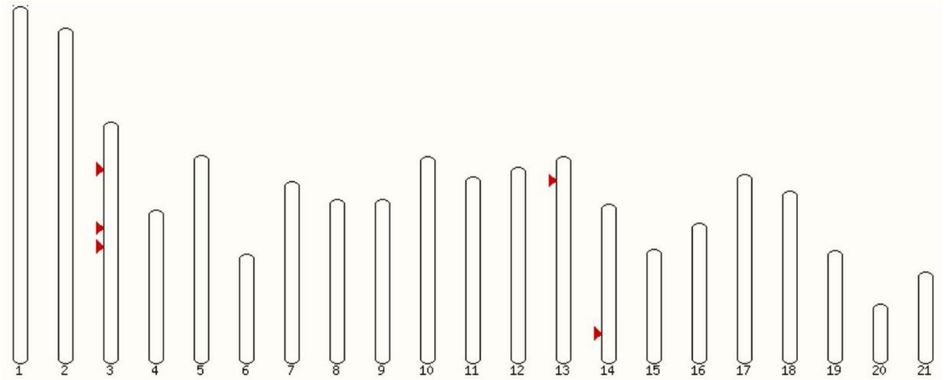

Figure 1. *Tetraodon nigroviridis*, karyogram with labeled 5S rDNA loci (red arrowheads). Note: this species represents one of the smallest fish genomes (C-value, the haploid genome size C ≈ 0.4) and correspondingly low number of 5S rDNA sites was detected and visualized (of the 80 5S rDNA sites identified by BioMart only five could be assigned to a linkage group and localized on chromosomes).

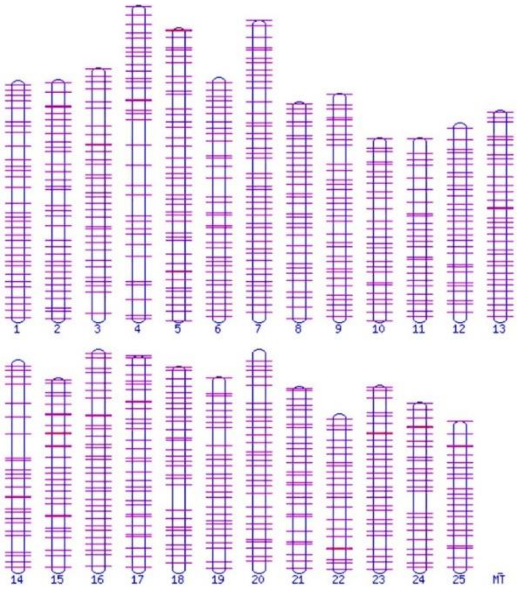

Figure 2. Zebrafish (*Danio rerio*), complete karyogram with labeled 5S rDNA loci (pink lines). Note: this species represents one of the largest genomes among teleost fish (C ≈ 1.7). Hence, a correspondingly high number of 5S rDNA sites was detected and visualized.

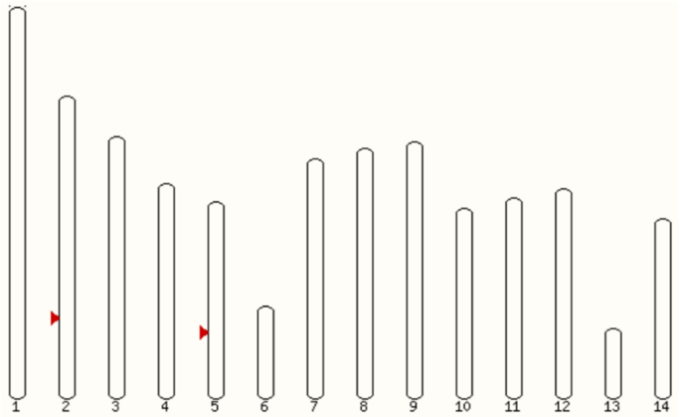

Figure 3. A tunicate sea squirt (*Ciona intestinalis*), C ≈ 0.2, the thirty-five 5S rDNA site detected on four chromosomes could be assigned to a linkage group.

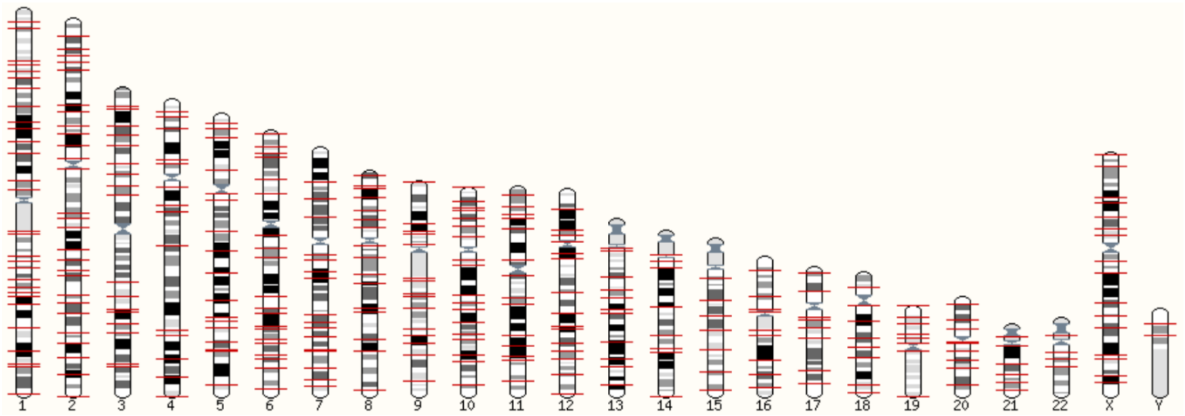

Figure 4. Human genome illustrates here the best assembled vertebrate genome, C ≈ 3.5. Note: in contrast to fish genomes, the chromosome morphology, i.e. the centromere position, is resolved in human genome, and the GC- and gene rich (black bands), AT-rich and gene poor (white bands), and intermediate regions (grey bands) are discriminated.
